# PTQ10:L8-BO organic photoactive layers enable improved stability for solar water oxidation and enhanced unassisted water splitting

**DOI:** 10.1039/d6el00052e

**Published:** 2026-03-20

**Authors:** Matyas Daboczi, Noof Al Lawati, Katherine Stewart, Maoqing Zhi, Jolanda Simone Müller, Ji-Seon Kim, Jenny Nelson, Flurin Eisner, Salvador Eslava

**Affiliations:** a Department of Chemical Engineering and Centre for Processable Electronics, Imperial College London London SW7 2AZ UK matyas.daboczi@ek.hun-ren.hu s.eslava@imperial.ac.uk; b HUN-REN Centre for Energy Research, Institute of Technical Physics and Materials Science Budapest 1121 Hungary; c Department of Physics and Centre for Processable Electronics, Imperial College London London SW7 2AZ UK jenny.nelson@imperial.ac.uk f.eisner@qmul.ac.uk; d Department of Materials, Imperial College London London SW7 2AZ UK; e School of Engineering and Materials Science, Queen Mary University of London E1 4NS UK

## Abstract

Integrating organic photovoltaics into anodes (IPV-anodes) represents a promising way to exploit the excellent optoelectronic properties of organic polymer: non-fullerene bulk-heterojunctions (BHJ) for solar-to-fuel applications. However, the high voltage losses, poor photochemical stability and high synthetic complexity of the most commonly used polymer: non-fullerene combinations have limited their full potential. Here, we address these limitations by introducing a BHJ comprising the low-synthetic-complexity polymer PTQ10 and the near-infrared absorbing acceptor L8-BO. By integrating this new BHJ with a graphite sheet functionalised with a NiFeOOH catalyst, we achieve a low onset potential of +0.64 V_RHE_, a photocurrent density of 21 mA cm^−2^ at +1.23 V_RHE_ and a *t*_80_ operational stability of 22 h under full AM1.5 G illumination (*i.e.*, without using any UV filter) for water oxidation. These values represent a 40 mV increase in photovoltage and a sevenfold improvement in operational stability (*t*_80_ extended from 3 h to 22 h) compared to reference IPV-anodes based on the ternary D18:PM6:L8-BO photoactive blend. Spectroscopic analyses reveal that these improvements stem from the reduced non-radiative voltage losses (from 0.24 V to 0.19 V) and superior photochemical and morphological stability of the PTQ10:L8-BO blend compared to the reference blend. Building on these advances, we demonstrate monolithic tandem IPV-anodes integrating PTQ10:IDIC and PTQ10:L8-BO organic blends to achieve a solar-to-hydrogen efficiency of 6.2%, offering critical insights for boosting the stability and efficiency of integrated solar-to-hydrogen systems working without any external bias.

## Abstract 2

PTQ10:L8-BO organic photoactive layers enhance performance and stability of integrated photovoltaic IPV-anodes, delivering 21 mA cm^−2^, *t*_80_ of 22 h under full solar irradiation (no UV filter), and >6% bias-free STH efficiency in tandem configurations.

## Graphical abstract

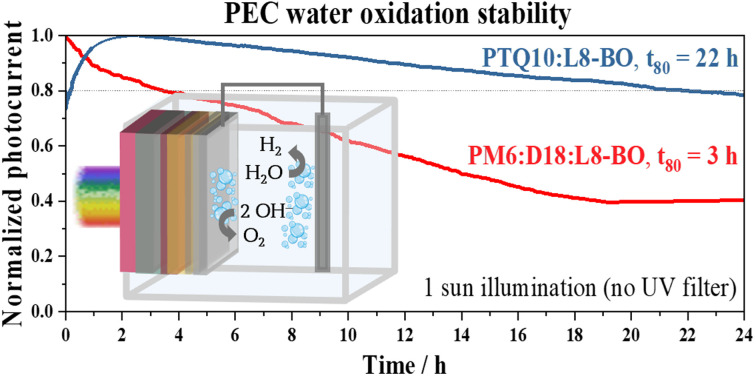

**Broader context**

Green hydrogen is a clean, renewable fuel that can store solar energy and help reduce our reliance on fossil fuels, which is key to reaching net-zero carbon emissions. A sustainable way of producing green hydrogen is through solar-driven electrochemical water splitting, leveraging advances in photovoltaics and photoelectrochemistry. Organic semiconductors offer exciting potential for these systems due to their low cost, tuneable properties, and scalable production. However, two key challenges hold back their practical use: poor stability under full-spectrum sunlight and insufficient voltage to efficiently drive water splitting. In this study, we show progress in both areas by developing anodes that integrate a PTQ10:L8-BO organic blend, which shows exceptional morphological and chemical stability. This allows the devices to operate far longer than previously, even under full solar illumination conditions (*i.e.*, without using UV filter). The improved device also produces higher voltages, enabling the demonstration of a new record solar-to-hydrogen (STH) efficiency of 6.2% when used in a tandem configuration. Our findings mark a major step toward viable, stable, and high-performing organic integrated systems and offer a clear path forward for their integration into renewable hydrogen production technologies.

## Introduction

A promising approach to the mismatch of variable renewable power supply with electricity demand involves storing solar energy in the form of hydrogen (H_2_) through water splitting, enabled by semiconductor-based technologies. These systems include: photocatalytic (PC) reactors, photoelectrochemical (PEC) cells, and photovoltaic–electrochemical (PV–EC) systems.^1,2^ PV–EC is a technologically mature solution, however it requires sophisticated power electronics and is mostly suited for centralized infrastructure. In contrast, PC systems offer a more straightforward design, utilizing semiconductor particles and co-catalysts in direct contact with water within a single compartment, but current systems are limited by side-reactions, inefficient charge carrier separation and instability in the aqueous environment.^3^ PEC cells represent an intermediate solution, integrating semiconductors and electrocatalysts into photoanodes and photocathodes placed in separate compartments. This configuration enables the application of advanced layer-by-layer thin-film PV technologies for improved charge separation and allows greater control over the reaction environment and material stability. PEC cells have traditionally employed metal oxide semiconductors such as TiO_2_, α-Fe_2_O_3_, Cu_*x*_O and BiVO_4_ incorporating photoelectrodes with direct semiconductor–electrolyte interface to drive water oxidation and reduction.^4–8^ In recent developments, devices have integrated PV materials such as halide perovskites and electrocatalysts into monolithic electrode structures without a direct semiconductor–electrolyte interface.^9–12^ These fully integrated electrodes leverage advances in PV–EC while maintaining the relatively low complexity, cost-effectiveness and low current densities of traditional PEC cells, avoiding the need for power electronics and offering the potential for thermal integration.^2,13,14^ To distinguish these integrated electrodes from traditional photoelectrodes that operate *via* a direct semiconductor–electrolyte interface, as well as from non-illuminated anodes, we recently introduced the term integrated photovoltaic anode (IPV-anode).^15^

Organic semiconductors (OSCs) have emerged as attractive photoactive materials for solar hydrogen production because they are low-cost, comprise Earth-abundant elements, and are suitable for large-scale solution-processing.^16,17^ OSCs have tuneable optical and electronic properties, and their complete bandgap tuneability could theoretically allow for solar-to-hydrogen (STH) efficiencies exceeding 30% in PEC solar water splitting.^18,19^ The most efficient organic photoactive layers comprise a blend (termed a bulk-heterojunction, BHJ) of a polymer ‘donor’ and a small molecule or polymer ‘acceptor’, which allows for almost 100% absorbed photon-to-charge quantum conversion efficiency, from ultra-violet to near-infrared photon energies.^20^ Such BHJ blends have thus been used in the best performing OSC-based devices.^21–23^ For example, through the integration of a co-catalyst, Cho *et al.* demonstrated an all-polymer BHJ organic photoanode, achieving photocurrent density (*j*_ph_) over 2 mA cm^−2^ at +1.23 V *versus* the reversible hydrogen electrode (V_RHE_) for solar water oxidation.^24^ Through tuning the energy levels of the donor polymer, Sekar *et al.* achieved *j*_ph_ of 4.1 mA cm^−2^ at +1.23 V_RHE_ with BHJ organic photoanodes,^25^ whilst Zhang *et al.* developed an all-polymer BHJ photoanode with *j*_ph_ exceeding 4 mA cm^−2^ at 0 V_RHE_ and pH 9.^26^

Yu *et al.* employed a BHJ layer protected with GaIn eutectic and Ni foil. These IPV-anodes retained 90% of their initial *j*_ph_ of 15.1 mA cm^−2^ after 10 h operation at pH 14 and +1.23 V_RHE_, applying a 420 nm cut-off UV filter.^19^ Most recently, we fabricated IPV-anodes comprising a PM6:D18:L8-BO ternary BHJ photoactive layer protected by a graphite sheet functionalised by a NiFeOOH electrocatalyst, which successfully demonstrated that translating both the almost 100% absorbed-photon to charge conversion in organic photovoltaics (OPVs) and the high *j*_ph_ (>20 mA cm^−2^) to IPV-anodes driving solar water oxidation is feasible. These PM6:D18:L8-BO IPV-anodes reached days-long operational stability at pH 14 (*t*_80_ of 23 h, where *t*_80_ is the time it maintains 80% of its initial maximum *j*_ph_). However, this stability required applying a UV filter, which reduced the *j*_ph_ by ∼10%. Under full AM1.5 G illumination (*i.e.,* with no UV filter), these PM6:D18:L8-BO IPV-anodes showed limited stability (*t*_80_ of only 3 h). Nevertheless, this approach allowed the fabrication of monolithic tandem organic IPV-anodes with STH efficiency up to 5%, by adding a wide-bandgap BHJ sub-cell and a Pt counter-electrode.^15^ The integration of all the PV layers into the anode also simplified the optical management of the overall STH device, since light only needs to propagate through the tandem PV stack.^15^

These works demonstrate the strong potential of organic BHJ-based STH systems. However, two critical challenges remain: (1) the high photovoltage losses of organic BHJ photoactive layers relative to their optical bandgap,^27^ which limits the achievable turn-on voltage of IPV-anodes and thus the performance of devices for unassisted (*i.e.,* bias-free) solar water splitting; (2) the chemical and morphological instability of the most efficient BHJ layers under full AM1.5 G illumination.^28^ Here, we address these limitations through the introduction of a new BHJ blend based on the synthetically simple and photochemically stable polymer PTQ10,^29^ whose low synthetic complexity additionally increases industrial viability through lower manufacturing costs.^30^ By replacing the synthetically complex polymers D18 and PM6 in the L8-BO-based photoactive blend with PTQ10, we can simultaneously decrease photovoltage losses and enhance the operational stability for water oxidation under full AM1.5 G illumination of IPV-anodes comprising PTQ10:L8BO BHJ layers and NiFeOOH catalyst-functionalised graphite sheets. We show that a reduction in photocurrent onset potential (*E*_on_) to +0.64 V_RHE_ in these IPV-anodes is enabled by the lower non-radiative voltage losses of the PTQ10:L8-BO blend (0.19 V), and that the record operational water oxidation stability of *t*_80_ = 22 h is due to improved photochemical and morphological stability of the PTQ10:L8-BO BHJ layer. Finally, incorporating the narrow-bandgap PTQ10:L8-BO with a wide-bandgap PTQ10:IDIC photoactive layer into a monolithic tandem organic IPV-anode leads to an *E*_on_ of −0.25 V_RHE_ and unassisted (*i.e.*, bias-free) water splitting with a Pt counter-electrode at *j*_ph_ above 5 mA cm^−2^, achieving a new benchmark STH efficiency of 6.2%.

## Results and discussion

### Composition of single-junction and tandem organic IPV-anodes

**Fig. 1:**
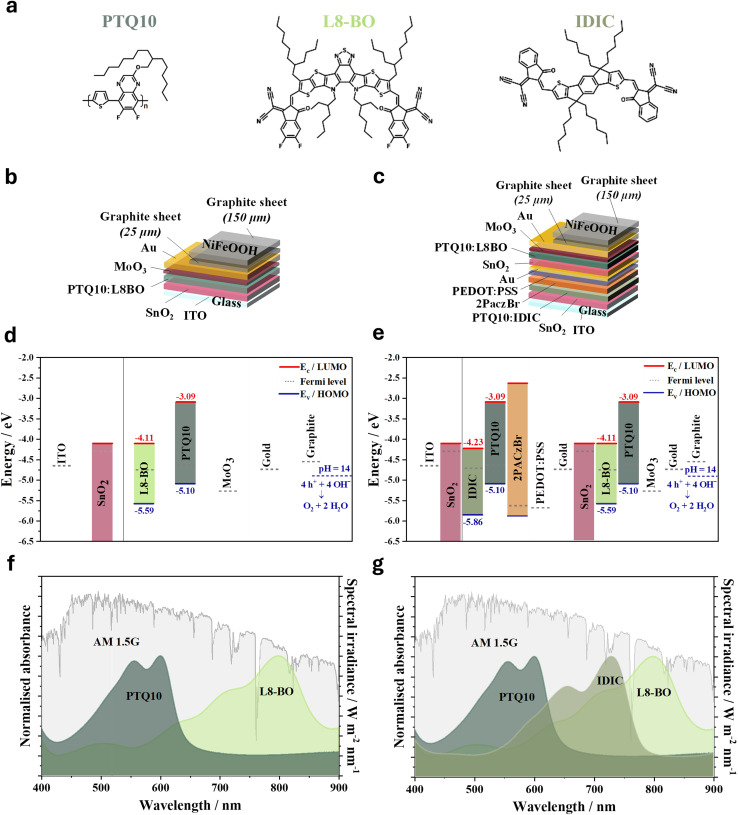
Structure, composition and energetics of the PTQ10:L8-BO single- and multi-junction organic IPV-anodes. (a) Chemical structures of the photoactive materials PTQ10, L8-BO and IDIC constituting the single- and multi-junction organic devices. Schematic representation of the inverted structure (b) single-junction and (c) tandem organic IPV-anode. Due to the direct electrical contact between the Au and rough graphite layers, the adhesive layer between them is not shown. Energy level diagrams for all constituent layers of the (d) single-junction and (e) tandem organic IPV-anodes. The electrochemical potential of water oxidation to oxygen at pH 14 is indicated by blue dashed lines on the absolute energy scale (shifted by 4.44 eV compared to the standard hydrogen electrode scale). Normalised absorbance spectra of the photoactive layers present in the (f) single-junction and (g) tandem organic devices relative to the AM 1.5 G standard solar spectrum.

Single-junction OPVs and corresponding single-junction organic IPV-anodes were prepared through sequential deposition of a SnO_2_ electron transport layer, a BHJ blend of PTQ10 polymer donor and L8-BO NFA,^31–33^ and evaporated MoO_3_ hole transport layer (Fig. 1, details in the Experimental Section in SI). The OPV devices were finished with a thin (40 nm) evaporated layer of Au, while the IPV-anodes were completed by adding the combination of a dense, 30 µm-thick self-adhesive graphite sheet and a more porous and thicker (150 µm) self-adhesive graphite sheet functionalised with a NiFeOOH catalyst, adapted from our previous work.^15^ Although the self-adhesive graphite contains an adhesive layer to ensure mechanical stability, the graphite still forms a direct ohmic contact with the Au layer, resulting in negligible electronic losses at the interface, as previously demonstrated.^15^

We chose PTQ10 to replace D18 and PM6 from our previous work in a BHJ blend with L8-BO due to a combination of its: (i) deeper highest occupied molecular orbital (HOMO),^34^ which reduces its energetic offset with L8-BO and likely leads stronger local excited (LE) – charge-transfer (CT) state mixing in the blend, which may reduce non-radiative voltage losses;^35,36^ (ii) superior photochemical stability under light and oxygen exposure compared to polymers containing the BDT-thiophene-motif (*e.g.* PM6 and D18);^37–40^ (iii) simpler synthesis (and hence lower cost and energy footprint); (iv) compatibility with more eco-friendly solvents, required for large-scale processing, in particular when combined with L8-BO, whose branched side-chains similarly allow processing with more benign solvents.^41^

Using air photoemission spectroscopy, we measured the HOMO of deposited PTQ10 films to be −5.10 eV, deeper than the HOMO of both D18 and PM6 (−5.05 eV) and the electrochemical water oxidation potential at pH 14 (−4.90 eV)), which is necessary for efficiently driving the chemical reaction (Fig. 1d and e). The measured absorption spectra of PTQ10 and L8-BO (Fig. 1f) show that PTQ10 and L8-BO have complementary absorption, where PTQ10 primarily absorbs higher energy photons in the wavelength range of 450–650 nm, while the narrower bandgap L8-BO absorbs the near-infrared photons with peak absorption at around 800 nm.

In the tandem organic IPV-anode (demonstrated towards the end of the paper), the PTQ10:L8-BO device was used as the back sub-cell connected *via* an interconnecting layer (ICL) of BM-HTL-1, 1 nm Au, and SnO_2_ to the front sub-cell containing a photoactive layer of PTQ10:IDIC BHJ blend. IDIC was chosen as the NFA in the front sub-cell due to its absorption peak at around 730 nm allowing for complementary absorption in a wide-bandgap component sub-cell in tandem with an PTQ10:L8-BO (Fig. 1c, e and g).^37^

### Performance of single-junction OPVs and IPV-anodes

**Fig. 2:**
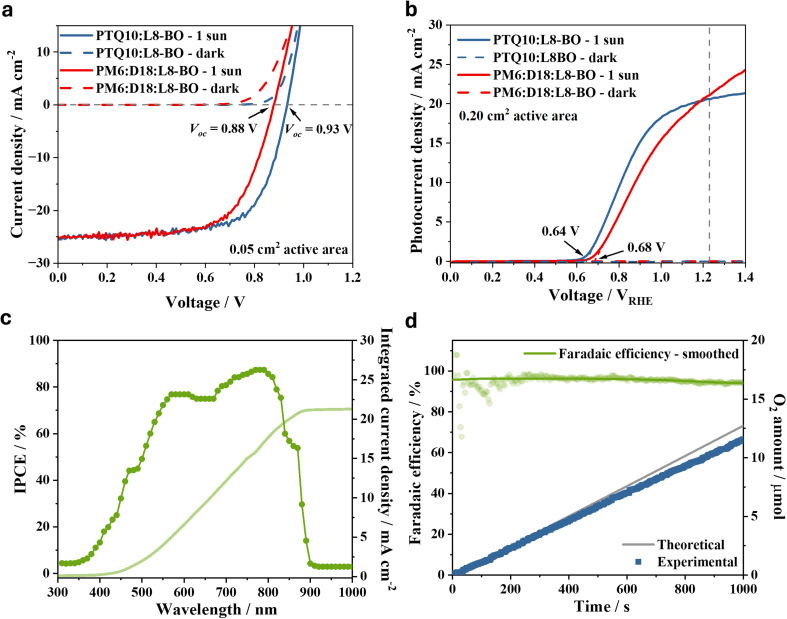
Performance of PTQ10:L8-BO single-junction organic solar cells and IPV-anodes. (a) Current density–voltage scans of the 0.05 cm^2^ active area PTQ10:L8-BO and the reference PM6:D18:L8-BO organic solar cells measured under 1 sun illumination and in dark at 0.1 V s^−1^ and 1 V s^−1^ scan rate, respectively. (b) Current density–voltage scans of the 0.2 cm^2^ active area PTQ10:L8-BO and PM6:D18:L8-BO organic IPV-anodes measured under 1 sun illumination and in dark at 0.05 V s^−1^ scan rate. The dashed vertical line indicates +1.23 V_RHE_ applied potential. (c) IPCE spectrum and integrated photocurrent density at +1.23 V_RHE_ of the PTQ10:L8-BO organic IPV-anode. (d) Faradaic efficiency of the PTQ10:L8-BO organic IPV-anode calculated from the experimentally determined amount of O_2_ compared to the theoretical amount of O_2_ based on the recorded photocurrent. The green circles represent each measurement point, while the green line represent the smoothed data. The measurements were performed in aqueous, 1 M NaOH electrolyte in three-electrode setup.

The current–voltage curves under AM1.5 G illumination of optimised PM6:D18:L8-BO reference and PTQ10:L8-BO single-junction OPV devices with an active area of 0.05 cm^2^ are shown in Fig. 2a. Both devices generate a short-circuit current density (*J*_sc_) of around 25 mA cm^−2^, however, the PTQ10:L8-BO devices display a 50 mV higher open circuit voltage (*V*_oc_, 0.93 V) compared to the PM6:D18:L8-BO device (0.88 V), which correlates with the 50 meV deeper HOMO of PTQ10 compared to PM6 and D18. To determine the origin of the higher *V*_oc_, we performed a voltage-loss analysis of the two devices using high-sensitive external quantum efficiency and electroluminescence measurement (Fig. S1 and Table S1), following our previously established procedure.^42^ We find that the higher *V*_oc_ of PTQ10:L8BO is entirely due to a reduction in the non-radiative voltage loss, which is 0.19 V in PTQ10:L8-BO devices compared to 0.24 V in PM6:D18:L8-BO devices, rather than to a change in the radiative limit to the open circuit-voltage (*V*_oc,rad_). This would be consistent with a higher energy and brighter interfacial CT state in PTQ10:L8-BO compared to PM6:D18:L8BO, due to increased mixing between CT and excited states at lower HOMO offset between the donor and acceptor.^35,36^ Interestingly, the decreased non-radiative voltage loss in PTQ10:L8-BO is not associated with a corresponding decrease in charge-generation efficiency (*i.e.* the EQE), as is usually the case for systems with low energetic offsets in the ionisation potentials of the donor and acceptor.^43^ Indeed, the non-radiative voltage loss achieved for PTQ10:L8-BO is amongst the lowest for high-performance (>16% power conversion efficiency) organic solar cells and demonstrates the potential of PTQ10-based solar cells to mitigate the known trade-off in organic solar cells of achieving both low photovoltage losses and high photo-charge generation efficiency.^44^

These organic photoactive layers were integrated into single-junction anodes with the aforementioned NiFeOOH catalyst-functionalised graphite sheet and tested for water oxidation in a three-electrode setup under continuous and chopped 1 sun illumination (Fig. 2b and S2). The *E*_on_ of the PTQ10:L8-BO IPV-anode was 40 mV lower (+0.64 V_RHE_) compared to the reference PM6:D18:L8-BO IPV-anode (+0.68 V_RHE_), which agrees with the higher photovoltage observed for the PTQ10:L8-BO OPV devices. This shows that reducing the non-radiative voltage loss of the organic photoactive layer through the modulation of the energy levels of the constituent semiconductors is an effective strategy to improve the *E*_on_ of IPV-anodes. *E*_on_ was determined conservatively by linear fitting of the photocurrent rise. The improved photovoltage of the PTQ10:L8-BO IPV-anode was confirmed by the 40 mV larger change in open circuit voltage (ΔOCP) upon switching from 1 sun illumination (AM1.5 G) to dark condition (Fig. S3). The *j*_ph_ at +1.23 V_RHE_ achieved was around 21 mA cm^−2^ for both organic IPV-anodes, which is lower than the 25 mA cm^−2^*J*_sc_ of the OPV devices likely due to the four times larger photoactive area used (0.2 cm^2^) leading to decreased shunt resistance and fill factor. This drop in *j*_ph_ is not caused by differences in the IPV-anode compared to OPV, but rather by the increased active area from 0.05 cm^2^ to 0.2 cm^2^, as confirmed by the *J*_sc_ decrease that is also observed in larger-area OPV (Fig. S11).^15^ Similar to our previous work investigating PM6:D18:L8-BO organic IPV-anodes, we found that a thicker photoactive layer is necessary to reach the highest performance with larger active area IPV-anodes, which is in agreement with the need of compensating a lower shunt resistance (Fig. S4).^15^ The high water oxidation *j*_ph_ generated by the PTQ10:L8-BO IPV-anode was confirmed by the incident photon-to-current efficiency (IPCE) at +1.23 V_RHE_ reaching above 80% at its maximum with a *j*_ph_ of 21 mA cm^−2^ calculated by integrating the product of IPCE, unit charge and the standard AM1.5 G solar spectrum (Fig. 2c). Oxidation of water into oxygen was validated through the measurement of an average faradaic efficiency of 96% using an O_2_ fluorescence sensor (Fig. 2d).

### Operational water oxidation stability of single-junction organic IPV-anodes

**Fig. 3:**
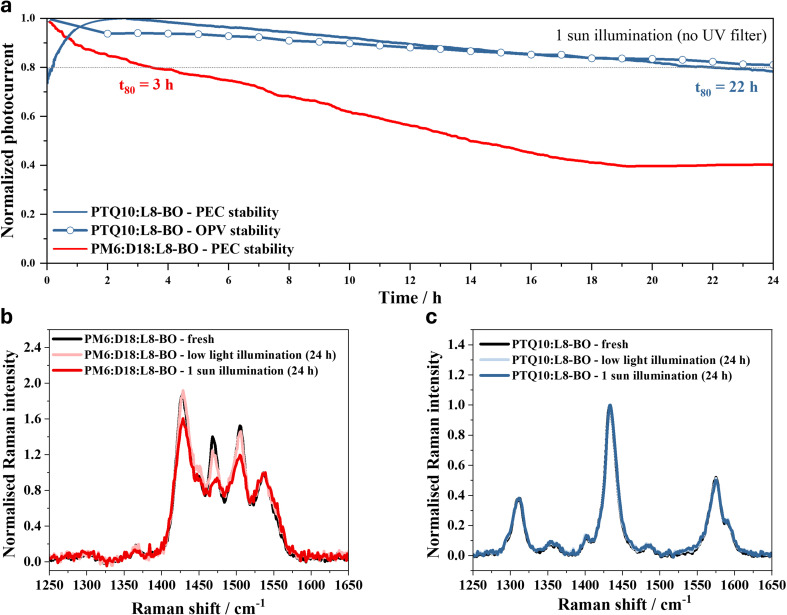
Operational stability of 0.2 cm^2^ active area PTQ10:L8-BO single-junction organic IPV-anodes and solar cells measured at ambient conditions, under 1 sun illumination without using any UV filter. (a) Operational water oxidation photocurrent stability at +1.23 V_RHE_ of the PTQ10:L8-BO and the reference PM6:D18:L8-BO IPV-anodes compared to the photocurrent stability of PTQ10:L8-BO solar cell at 0.2 V. The solar cell was kept at 0.2 V applied bias and current density–voltage scans were recorded once every hour. 1 M aqueous NaOH electrolyte was used for the measurements. The horizontal dashed line indicates 80% of the maximum initial photocurrent of the devices. (b) Raman spectra at 514 nm excitation of the PM6:D18:L8-BO and (c) PTQ10:L8-BO photoactive layers after 24 h illumination by low light (∼0.1 sun) and 1 sun, compared to fresh samples.

The organic IPV-anodes were tested for continuous operational water oxidation stability under full simulated solar illumination (*i.e.*, without using any UV filters) at +1.23 V_RHE_. The PTQ10:L8-BO device showed a *t*_80_ of 22 h, where *t*_80_ is defined as the time until the IPV-anode retains 80% of its initial maximum photocurrent density, which is seven times longer than the *t*_80_ of 3 h recorded for the PM6:D18:L8-BO reference device (Fig. 3a). We note that the *t*_80_ stability lifetime is an accepted standard for stability measurements in photovoltaics, and we encourage its adoption for PEC-related stability measurements. Furthermore, the PTQ10:L8-BO IPV-anode remained operational (*i.e.,* no catastrophic failure) for water oxidation for more than 2 days (Fig. S5).

In order to understand the superior stability of the PTQ10-containing organic photoactive layer, the degradation of thin films of PTQ10:L8-BO and PM6:D18:L8-BO deposited on glass/ITO/SnO_2_ substrates was investigated at ambient condition under illumination by different light intensities. Raman spectroscopy showed no change for the PTQ10:L8-BO layer neither by low light (∼0.1 sun) nor by 1 sun intensity full solar 24 h long illumination in air (Fig. 3c). In contrast, for the PM6:D18:L8-BO films, there was a clear decrease in the delocalised backbone peak intensity of both donor polymers already after illumination with low light intensity (Fig. 3b). At low light, there is a decrease in the delocalised C=C peak at 1470 cm^−1^, and at 1 sun the quenching is amplified and extended to the C–C mode at 1429 cm^−1^ (Raman peak assignment in Fig. S6). This was previously linked to the photodegradation of PM6 and D18 polymers through increased torsion between the donor and acceptor units, reducing polaron delocalisation along the backbone.^29^ Additionally, at 1 sun illumination there is a prominent relative peak intensity decrease of the D18 only peak (1505 cm^−1^) while the PM6 only peak (1450 cm^−1^) remains unchanged. This suggests that D18 has a greater conformational instability under photodegradation than PM6. This donor backbone conformational change is in agreement with clear loss of vibronic structure of the polymer absorbance band in the PM6:D18:L8-BO film after 24 h illumination even by low light intensity, while no observable change for the PTQ10:L8-BO blend (Fig. S7 and S8). Similarly, there was a red shift of the photoluminescence (PL) peak of the acceptor in the PM6:D18:L8-BO sample even at low light illumination, which we previously identified as a sign of morphological instability.^15^ On the other hand, there was no significant change in the PL spectrum of the PTQ10:L8-BO film at low light illumination and only a slight peak broadening at 1 sun illumination (Fig. S9 and S10). These results reveal that a reason behind the enhanced operational water splitting stability of the PTQ10:L8-BO IPV-anodes is the improved photochemical and morphological stability of the PTQ10:L8-BO BHJ blend. This can be rationalised by the light-induced dihedral twisting around the BDT-T motif of PM6 and D18, which is not present in PTQ10 and by the high molecular weight of PTQ10 used, which was shown to increase thermal stability.^29,45^

Although there was no observable change in the Raman spectra of PTQ10:L8-BO film, indicating excellent photochemical stability, the broadening of the PL spectrum of the thin film after 24 h full 1 sun illumination and the change in the UV-Vis spectrum suggest some degree of morphological instability (Fig. S8 and S10). This degradation pathway can be assigned to the instability of photoactive layer in the OPV device and not to the electrolyte/catalyst interface or deterioration of the catalyst, which is evidenced by the similar, linear photocurrent decay of the OPV and the IPV-anode within the first 24 h of operation (Fig. 3a and S11). After 24 h of continuous water oxidation, the *j*_ph_ decay rate increased significantly from −0.2 to −0.3 mA cm^−2^ h^−1^; however, the slower decay was recovered after replacing the graphite sheet with fresh graphite/NiFeOOH layer (Fig. S5) as previously. This result confirms that the slower linear *j*_ph_ decay of the IPV-anode (0–24 h operation) is due to the degradation of photoactive layer, and it also shows that the accelerated IPV-anode degradation (after 24 h operation) is due to the loss of catalyst resulting from the electrochemical oxidation and resulting disintegration of the graphite/NiFeOOH sheet (Fig. S12).

### Performance of tandem organic IPV-anode

To leverage the high photovoltage produced by the single-junction PTQ10:L8-BO IPV-anodes, we fabricated monolithic tandem organic IPV-anodes as previously (Fig. S13). The narrow-bandgap PTQ10:L8BO device served as the back sub-cell, while a wide-bandgap PTQ10:IDIC device formed the front sub-cell (Fig. 1), with BM-HTL-1/Au(1 nm)/SnO_2_ as ICLs.^27^ This tandem configuration shifted the water oxidation *E*_on_ from +1.54 V_RHE_ measured under dark condition for the graphite/NiFeOOH sheets to −0.25 V_RHE_ under 1 sun illumination in the IPV-anode (Fig. S14). This shift is in accordance with the *V*_oc_ of the tandem OPV (approx. 1.8 V), and with the ΔOCP of the IPV-anodes (approx. 1.6 V) (Fig. S15 and S16). The shifted, negative *E*_on_ of the tandem organic IPV-anode allowed for *j*_ph_ generation up to 6.6 mA cm^−2^ at 0 V_RHE_ both under continuous and chopped illumination (Fig. S17 and S18). This performance was achieved by optimising the composition of ICLs for efficient charge separation with minimal optical losses and low electrical resistance, which was found optimal with BM-HTL-1/Au(1 nm)/SnO_2_ (Fig. S19).^46^

**Fig. 4:**
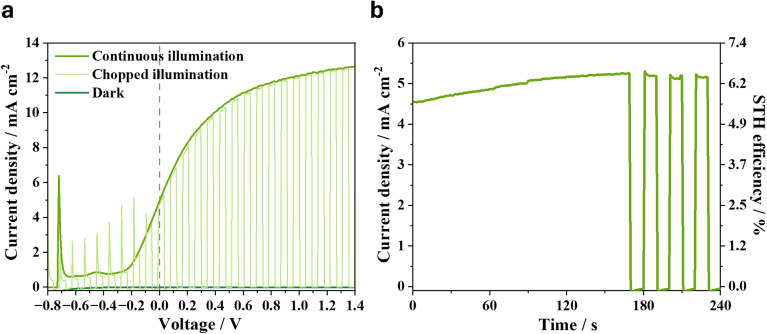
Performance of monolithic tandem organic IPV-anodes incorporating PTQ10:L8-BO and PTQ10:IDIC photoactive layers. (a) Current density–voltage scans of the tandem organic device measured in a two-electrode setup in dark, under 1 sun continuous and chopped illumination at 0.05 V s^−1^ scan rate. (b) Unassisted photocurrent stability of the organic tandem IPV-anode measured in a two-electrode setup under 1 sun illumination, which was chopped after 170 s of continuous illumination. The measurements were performed in aqueous, 1 M NaOH electrolyte.

The PTQ10:L8-BO + PTQ10:IDIC tandem IPV-anode was also tested in a two-electrode setup under bias-free condition and achieved a *j*_ph_ of 5 mA cm^−2^ under continuous and chopped 1 sun illumination (Fig. 4a). In continuous unassisted solar water splitting operation the tandem organic IPV-anode reached a maximum *j*_ph_ of 5.2 mA cm^−2^, which translates into a 6.4% STH efficiency with 100% faradaic efficiency, or 6.2% STH efficiency considering the measured 96% faradaic efficiency for oxygen evolution (Fig. 4b). This is an important step forward compared to our previous record 5% STH efficiency reached with PM6:D18:L8-BO. We attribute this improvement to the reduced voltage losses allowing for enhanced photovoltage while maintaining high *j*_ph_.^15^

The *j*_ph_ of the tandem IPV-anode decayed to below 1 mA cm^−2^ after 3 h of continuous unassisted solar water splitting operation in a two-electrode setup (Fig. S20). Given the operational water oxidation stability of the single-junction PTQ10:L8-BO IPV-anodes (*t*_80_ = 22 h, Fig. 3), the fast decay observed in the tandem device plausibly originates from the front sub-cell, which contains the wide-bandgap PTQ10:IDIC BHJ blend. This blend has previously shown instability, which has been attributed to photoisomerization of IDIC, even when exposed only to white LED light.^47^

Interestingly, the tandem IPV-anode exhibited minimal *j*_ph_ decay at high applied potentials such as +1.23 V_RHE_ (Fig. S21), typically used to characterize photoanodes of low photovoltage developed for integration in tandem devices with photocathodes.^15,19^ A high applied potential such as +1.23 V_RHE_ in a three-electrode system is far from the 0 V_RHE_ operating point and from the bias-free two-electrode condition, where degradation-related shifts in *E*_on_ or fill factor are most directly observable. This highlights the importance of testing stability at relevant operating points and configurations, specifically in a two-electrode setup at zero applied bias when targeting unassisted solar water splitting, where even small changes caused by the degradation of the catalyst and/or photoactive layer will cause voltage losses and concomitant operating *j*_ph_ decay. These results suggest that future work should primarily focus on improving the photostability of the wide-bandgap BHJ layer, along with the long-term stability of the graphite catalyst sheet, in order to translate the promising stability of the PTQ10:L8-BO devices into tandem organic IPV-anodes.

## Conclusions

In this contribution, we presented organic integrated photovoltaic anodes (IPV-anodes) based on an organic BHJ blend comprising the synthetically simple polymer donor PTQ10 and the near-infrared absorbing non-fullerene acceptor L8-BO. By integrating the PTQ10:L8-BO blend as the photoactive layer with NiFeOOH functionalised protective sheets, we demonstrate monolithic IPV-anodes with a low water oxidation photocurrent onset potential of +0.64 V_RHE_ alongside high photocurrent density above 20 mA cm^−2^ at +1.23 V_RHE,_ and sevenfold enhanced continuous water oxidation stability (*t*_80_ of 22 h) under continuous full 1 sun illumination compared to the previous state-of-the-art PM6:D18:L8-BO-based devices (*t*_80_ of 3 h). By studying the voltage losses in PTQ10:L8-BO, we attribute the low onset potential to the low-non-radiative voltage loss of 0.19 V of this blend, amongst the lowest reported for high-efficiency polymer: non-fullerene photoactive layers. Raman, UV-Vis and photoluminescence spectroscopy studies, alongside comparisons of solar cell and IPV-anode stabilities, indicate that the improved operational stability is due the superior photochemical and morphological stability of the PTQ10:L8-BO organic bulk heterojunction layer. Finally, we fabricated monolithic tandem devices through the incorporation of a wide-bandgap PTQ10:IDIC front sub-cell, leading to IPV-anodes with a photocurrent onset potential of −0.25 V_RHE_ and an unassisted water splitting solar-to-hydrogen efficiency of 6.2%. We show that the operational stability of these tandem devices is limited by the photostability stability of the wide-bandgap BHJ layer and the long-term degradation of the protective graphite/catalyst sheet, pointing towards tangible pathways to improving the performance of these monolithic IPV-anodes towards efficient, stable and green hydrogen production.

## Author contributions

M. D., F. E., J. N. and S. E. conceived, designed, and supervised the project. N. A. L., F. E., and M. Z. fabricated the organic solar cells and IPV-anodes. The photovoltaic characterisation was implemented by N. A. L. and F. E., while N. A. L. and M. D. performed the irradiated electrochemical measurements. K. S. contributed by recording the Raman spectra. UV-Vis and energy level measurements were performed by N. A. L. and M. D. J. S. M performed the EL and EQE measurements. The manuscript was written by N. A. L. and M. D. with support from F. E., K. S, J. N. and S. E. All authors contributed to data analysis discussion of the results.

## Conflicts of interest

The authors declare no conflicts of interest.

## Supplementary Material

EL-002-D6EL00052E-s001

## Data Availability

The data that support the findings of this study are openly available in the following Figshare data repository at DOI: https://doi.org/10.6084/m9.figshare.31843918. Supplementary information (SI) is available. See DOI: https://doi.org/10.1039/d6el00052e.
