# Supplementary material for: PTQ10:L8-BO organic photoactive layers enable improved stability for solar water oxidation and enhanced unassisted water splitting
Source: EES Solar. 2026 Mar 20;2(3):677–85. doi: 10.1039/d6el00052e (PMC13019855; doi:10.1039/d6el00052e)
Supplement: EL-002-D6EL00052E-s001 [file EL-002-D6EL00052E-s001.pdf]

Supplementary Information (SI) for EES Solar.
This journal is © The Royal Society of Chemistry 2026

Supporting Information

PTQ10:L8-BO Organic Photoactive Layers Enable Improved Stability for Solar Water Oxidation and Enhanced Unassisted Water Splitting

Matyas Daboczi,[*ab†] Noof Al Lawati,[ac†] Katherine Stewart,[c] Maoqing Zhi,[ac] Jolanda Simone Müller,[c] Ji-Seon Kim,[c] Jenny Nelson,[*c] Flurin Eisner,[*cde] Salvador Eslava[*a]*

[a] Department of Chemical Engineering and Centre for Processable Electronics, Imperial College London, London SW7 2AZ, UK

[b] HUN-REN Centre for Energy Research, Institute of Technical Physics and Materials Science, Budapest, 1121, Hungary

[c] Department of Physics and Centre for Processable Electronics, Imperial College London, London SW7 2AZ, UK

[d] Department of Materials, Imperial College London, London SW7 2AZ, UK

[e] School of Engineering and Materials Science, Queen Mary University of London, E1 4NS, UK

*e-mail: matyas.daboczi@ek.hun-ren.hu; jenny.nelson@imperial.ac.uk; f.eisner@qmul.ac.uk; s.eslava@imperial.ac.uk

† Equal contribution authors.

Supplementary Information

Methods

Materials. Poly[[6,7-difluoro[(2-hexyldecyl)oxy]-5,8-quinoxalinediyl]-2,5-thiophenediyl]] (PTQ10) with a molecular weight of 62 kDa as measured by the supplier, 2,2'-((2Z,2'Z)-((12,13-bis(2-ethylhexyl)-3,9-(2-butyloctyl)-12,13-dihydro-[1,2,5]thiadiazolo[3,4-e]thieno[2",3'':4',5']thieno[2',3':4,5]pyrrolo[3,2-g]thieno[2',3':4,5]thieno[3,2-b]indole-2,10-diyl)bis(methanylylidene))bis(5,6-difluoro-3-oxo-2,3-dihydro-1H-indene-2,1-diylidene))dimalononitrile (L8-BO) with molecular weight of 1479.98 g mol^{-1}, and 2,2'-((2Z,2'Z)-((4,4,9,9-tetrahexyl-4,9-dihydro-s-indaceno[1,2-b:5,6-b']dithiophene-2,7-diyl)bis(methanylylidene))bis(3-oxo-2,3-dihydro-1H-indene-2,1-diylidene))dimalononitrile (IC-C6IDT-IC or IDIC) with molecular weight 1011.39 g mol^{-1} were purchased from Ossila. 1,4-Diiodobenzene additive was purchased from Sigma-Aldrich with a molecular weight of 329.90 g mol^{-1}. Nickel sulfate hexahydrate (Sigma Aldrich, ≥98%) and iron sulfate heptahydrate (Sigma Aldrich, ≥99%) were used for catalyst NiFeOOH preparation. Graphite sheets of different thickness, 25 µm thick (labelled G3) and 150 µm thick (labelled G15), were both purchased from RS Components Ltd, for protection and catalyst deposition, respectively. 2-propanol (IPA) and acetone for film cleaning were purchased from Sigma Aldrich. Decon90 was purchased from Decon Laboratories Ltd as the detergent used for cleaning substrates. Chloroform ($CHCl_3$) solvent was purchased from Sigma Aldrich, ≥99%. All solvents used for device fabrication were anhydrous and purchased from Sigma Aldrich, unless stated otherwise. BM-HTL-1 (an ethanol-based PEDOT:PSS formulation) was purchased from Brilliant Matters Inc. HTL Solar 1 (an

aqueous PEDOT:PSS formulation) was purchased from Ossila. 2PACz-Br was purchased from TCI.

OPV vs. IPV-anode devices. Organic solar cells were fabricated with an inverted device architecture. Centred-ITO (7-9 Ω resistance) glass substrates were used to fabricate OPV devices, whilst sided-ITO (7-9 Ω resistance) glass substrates were used to fabricate monolithic IPV-anodes. Two shadow masks were used for the deposition of the top contact on OPV devices resulting in a pixel area of 0.05 or 0.20 cm^2. In contrast, no shadow mask was used for devices intended as IPV-anodes. OPVs were prepared using a solution of 15 mg mL^{-1} of PTQ10:L8-BO (1:1.2 ratio), 50wt% of DIB additive, and chloroform. IPV-anodes were prepared with both 15 and 20 mg mL^{-1}, being optimal at 20 mg mL^{-1} resulting in a thickness of ~100 nm. **Table S2** summarises the optimum conditions used for the organic IPV-anodes. Further details are provided in the following sections.

Single-junction OPV. ITO glass substrates were sequentially cleaned by ultrasonication in detergent (Decon90), followed by deionised water, acetone, and IPA for 10 min respectively and then dried by nitrogen. The substrates were then treated by a UV ozone cleaner for 12 min to ensure clean surfaces before spin-coating. The substrates were then transferred to a nitrogen-filled glovebox for all the following thin-layer deposition steps. Tin oxide (SnO_2) as ETL was dynamically spin-coated (35µL) on the substrates at 2000 rpm for 35 s from a butanol mixture (Avantama N-30, 2.5 wt% of crystalline SnO_2), followed by thermal annealing (TA) for 30 min at 210°C on a hotplate before deposition of the active layer. PTQ10 and L8-BO were blended in a 1:1.2 ratio with 50wt% of DIB additive, and chloroform solvent was added to form a total concentration of 15 mg mL^{-1}. Solutions were heated at 50°C for 2.5 h before the deposition. The active layer PTQ10:L8-BO was dynamically spin-coated (35 µL) onto the first SnO_2 layer at 3000 rpm for 45 s followed by TA at 95°C for 5 min. 7 nm of MoO_3 as HTL and 40 nm of Au layer as top contact were both thermally evaporated under high vacuum (10^{-6} mbar).

Tandem OPV.

Similar fabrication steps as those for the single-junction OPV were followed, with the addition of various steps for the wide-bandgap (WBG) layer deposition and serial interconnection. Upon preparation of glass/ITO/SnO_2 samples, PTQ10 and IDIC were blended in a 1:1.5 ratio and chloroform solvent was added to form a total concentration of 30 mg mL^{-1}. Solutions were heated at 50°C for 1 h before the deposition. WBG PTQ10:IDIC was then dynamically spin-coated (35 µL) onto the SnO_2 top layer at 3500 rpm for 45 s followed by TA at 140°C for 5 min. Different ICLs were researched on top of the WBG PTQ10:IDIC layer, including 2PACz-Br, BM-HTL-1, HTL Solar 1 and different thicknesses of evaporated Au layer. For their deposition, samples with as-deposited and annealed WBG PTQ10:IDIC were taken out of the glovebox, pre-heated at 90°C, and spin-coated with either BM-HTL-1 (135 µL) statically at 3000 rpm for 1 min, HTL Solar 1 (135 µL) statically at 6000 rpm for 1 min, or 0.3 mg mL^{-1} 2PACz-Br in ethanol (15 µL twice) dynamically at 7000 rpm for 30 s. The 2PACz-Br sample was also followed by TA at 90°C for 2 min. All these samples were then placed back in the glovebox antechamber and pumped down for 30 min. Next, either no Au layer, or a 1 or 2 nm of Au layer was thermally evaporated under high vacuum (10^{-6} mbar) without shadow mask, forming the first sub-cell.

For the second sub-cell, SnO_2 as ETL was dynamically spin-coated (35 μL) onto the first sub-cell at 2000 rpm for 35 s and annealed at 100°C for 5 min. The next steps to complete the second sub-cell with narrow-bandgap (NBG) PTQ10:L8-BO, 7 nm of MoO_3, and 40 nm Au followed the exact same procedure discussed for a single-junction OPV.

Graphite catalytic sheets addition for IPV-anode fabrication. OPVs prepared on sided-ITO glass substrates, without shadow mask, and with both 15 and 20 mg mL^{-1} of PTQ10:L8-BO solutions were taken out of the nitrogen-filled glovebox to be completed for their application as IPV-anodes. The layers were scratched until a uniform ∼1 cm^2 area was reached where high resistance (1 kΩ – 1 MΩ) was measured from the ITO to the Au layer. A self-adhesive 25 μm thick graphite sheet (labelled G3) was manually applied on top of the ∼1 cm^2 delimited area, as a means of protection from water permeability and degradation. Finally, another self-adhesive 150 μm thick graphite sheet was applied on top, which was previously functionalised by NiFeOOH catalyst. NiFeOOH was electrodeposited by potential sweeps from –1.0 to +0.4 potentials applied versus Ag/AgCl reference electrode ($V_{Ag/AgCl}$) at 50 mV s^{-1} scan rate, in a three-electrode setup with Ag/AgCl reference and Pt counter electrode, using an aqueous solution of 40 mM nickel sulfate hexahydrate and 10 mM iron sulfate heptahydrate, previously purged with N_2 for 20 min. The electrodeposition was stopped when 25 mC cm^{-2} had been reached. A strip of adhesive Cu tape was applied to an exposed ITO area, and electrical contact was ensured using silver paste between the ITO and Cu tape. The steps of tandem organic IPV-anode fabrication are sketched in **Fig. S13**.

OPV characterisation. OPVs with 0.05 cm^2 pixel size were characterised by recording current density-voltage curves using a 4200 Keithley Source-Measure Unit integrated with an Oriel Instruments Solar Simulator equipped with a xenon lamp, under ambient air conditions. A Newport silicon photodiode was used for calibration to achieve AM1.5G equivalent to 100 mW cm^{-2} illumination intensity. External quantum efficiency (EQE) was measured with a Quantum Design PV300 system in air.

IPV-anode measurements. The IPV-anodes were tested as working electrodes in a three-electrode configuration with a Ag/AgCl reference electrode and Pt filament counter electrode, using an Ivium Compacstat potentiostat and aqueous 1 M NaOH electrolyte. IPV-anodes were illuminated by a Lot Quantum Design xenon lamp with AM1.5G filter through circular masks of area 0.197 cm^2 and 0.283 cm^2. International Light Technologies SEL623 photodetector was used for calibration to achieve AM1.5G equivalent of 100 mW cm^{-2} illumination intensity. Potentials applied versus Ag/AgCl reference electrode ($V_{Ag/AgCl}$) were converted to applied potential versus reversible hydrogen electrode (V_{RHE}) using the equation $V_{RHE} = V_{Ag/AgCl} + 0.0592 \times pH + 0.1976$, where 0.1976 V is the potential of the reference electrode versus the standard hydrogen electrode. Operational photocurrent stability measurements were performed at an applied bias of +1.23 V_{RHE} under continuous 1 sun illumination. These performance measurements were taken under ambient conditions in the lab. A two-electrode setup was used to measure tandem devices in unassisted, bias-free conditions, as well as their operational photocurrent stability. Faradaic efficiency was determined by measuring the amount of generated O_2 using a Pyroscience FireStingO2 fibre-optic oxygen meter with a TROXROB10 oxygen probe and compared to the theoretical amount of O_2 based on the recorded photocurrent.

Energy level measurements. Ambient photoemission spectroscopy (APS) was conducted using an SKP5050 (KP Technology) by scanning UV light irradiation within the range of 4.8-6.0 eV. The valence band edge (E_v) values of the thin layers were determined by extrapolating the cube root photoemission to zero. The Fermi level of the samples was calculated through contact potential difference measurements performed using a Kelvin probe, accounting for the work function of the vibrating tip. To calibrate the tip's work function, a freshly cleaned Ag reference was used, and its work function was determined via APS.

Optoelectronic characterisation. UV-Vis spectroscopy measurements were conducted on spin-coated solar cell materials on glass substrates (SnO_2, PTQ10, L8-BO, IDIC, MoO_3) by a Shimadzu UV-2600 spectrophotometer in transmittance mode. Electroluminescence (EL) spectra were obtained by an Andor iDUS InGaAs detector, in wavelength range from 600-1800 nm for 5 s exposure at different supplied currents 0.1, 0.2, 0.5, 1.0 and 2.0 mA. EL was fitted with EQE using a MATLAB software that follows the reciprocity relationship.

Photoluminescence and Raman spectroscopy. A Renishaw inVia Raman microscope was used to collect Raman spectra and with a 50x objective in a backscattering configuration. Spectra were collected using a 514 nm argon-ion laser; for PL spectra were recorded with no correction being applied for instrument response. Calibration of the filter and grating was performed using the well-defined 520 cm^{-1} peak of a Si reference. The sample was contained in Linkam THMS600 sample chamber under N_2 flow. Acquisition times and laser powers for both PL and Raman measurements were optimised to give the best spectra but were kept consistent between samples. For each sample and exposure condition, 5 spectra were taken across the whole sample area in order to account for variation and mean spectra displayed in figures. Raman peak assignment simulated using density functional theory (DFT) on the Imperial College High-Performance Computing service using GAUSSAIN16 software. All simulations were performed on single molecules in the gas phase using B3LYP level of theory and basis set 6-311G(d.p). Frequency of vibrations were identified from simulations of Raman spectra using empirical scaling factor of 0.97, and peak assignments were visualised using GaussView 6.0.16 software.[49]

Structural characterisation. Scanning electron micrographs were recorded at 5 kV accelerating voltage by a high-resolution field emission gun scanning electron microscope (LEO Gemini 1525). The thicknesses of the thin films were determined using a DektakXT stylus profilometer.

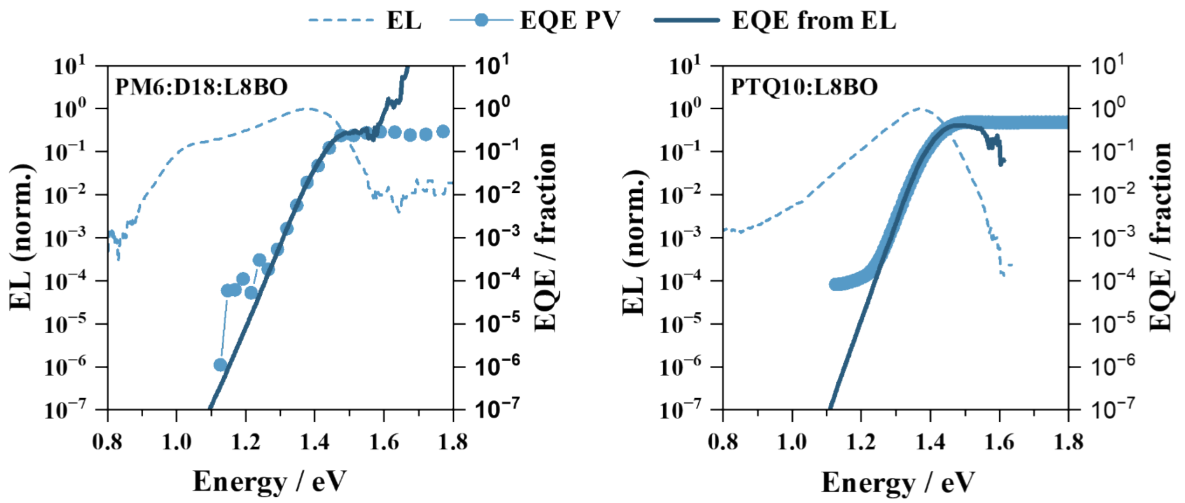

Fig. S1 Normalised electroluminescence (EL) and external quantum efficiency (EQE) spectra measured for the PM6:D18:L8-BO and PTQ10:L8:BO solar cells. Different injection currents provided the same results.

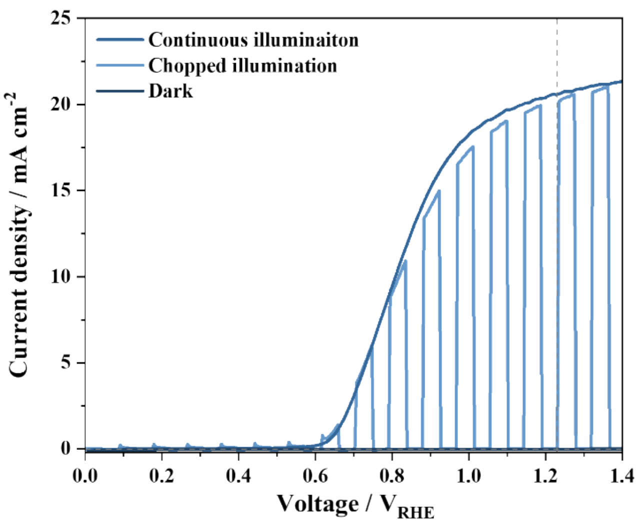

Fig. S2 Current density–voltage scans in dark, and under 1 sun continuous and chopped illumination for a PTQ10:L8-BO single-junction IPV-anode. Dashed vertical line indicates the applied potential of +1.23 V_{RHE}. The measurements were performed at 50 mV s^{-1} scan rate in an aqueous, 1 M NaOH electrolyte.

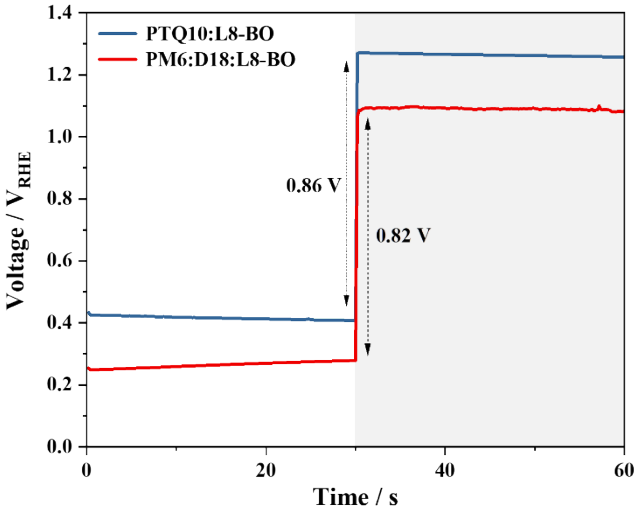

Fig. S3 Open circuit potential of the PTQ10:L8-BO and PM6:D18:L8-BO IPV-anodes measured under 1 sun illumination (0 – 30 s, white background) and in dark (30 – 60 s, shaded background) of the same devices. Dashed vertical lines with arrows indicate the difference in open circuit potential upon switching off the illumination. The measurements were performed in an aqueous, 1 M NaOH electrolyte.

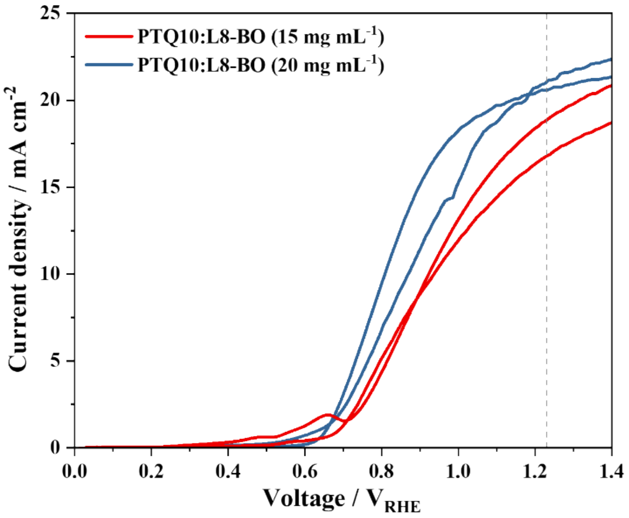

Fig. S4 Current density–voltage scans under 1 sun continuous illumination for PTQ10:L8-BO single-junction IPV-anodes prepared from different device batches with thinner and thicker active layers using precursor solutions with total concentrations of 15 and 20 mg mL^{-1}. Dashed vertical line indicates the applied potential of +1.23 V_{RHE}. The measurements were performed at 50 mV s^{-1} scan rate in an aqueous, 1 M NaOH electrolyte.

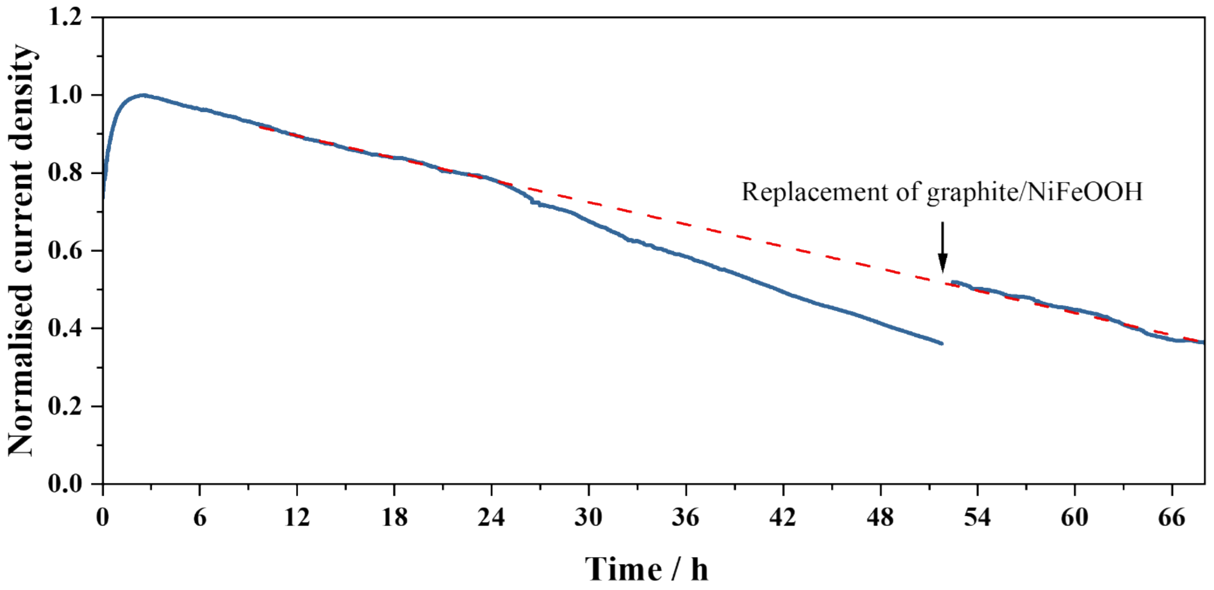

Fig. S5 Normalised operational photocurrent stability of a PTQ10:L8-BO organic IPV-anode measured in a three-electrode cell for 68 h at +1.23 V_{RHE}. The top 150 µm-thick graphite/NiFeOOH layer was replaced with a fresh one after 52 h of operation. Dashed red line shows linear fit for the most linear part (between 10–20 h) of the photocurrent decay. The measurement was performed in an aqueous, 1 M NaOH electrolyte.

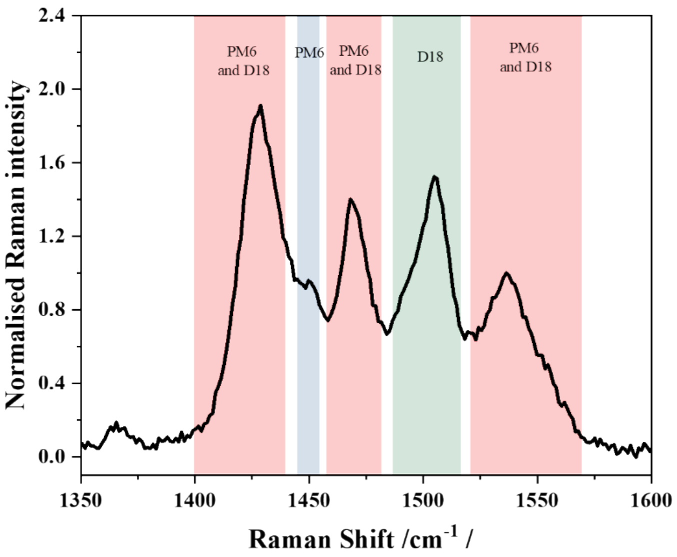

Fig. S6 Assignment of Raman peaks based on DFT calculations. The peaks at 1429 and 1468 cm^{-1} are attributed to delocalised vibrational modes along the whole conjugated backbone, with 1429 cm^{-1} assigned to the C-C single bond mode and 1468 cm^{-1} assigned to the C=C double bonds. The peak at 1536 cm^{-1} is assigned to the BDT core unit in both PM6 and D18. The peak at 1505 cm^{-1}

is assigned to the dithienobenzothiazole (DTBT) acceptor unit in D18. The peak at 1450 cm^{-1} is assigned to benzodithiophene dione (BDD) acceptor unit in PM6.

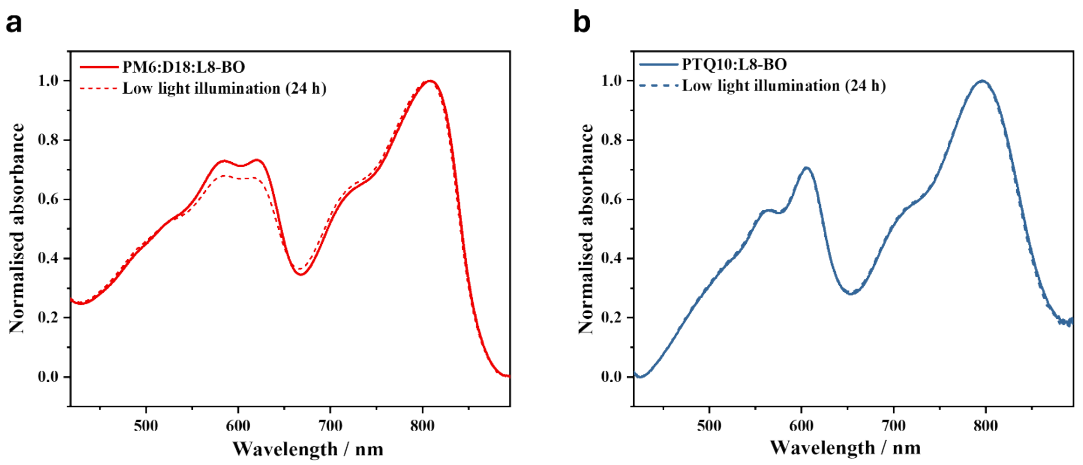

Fig. S7 Normalised UV-Vis absorbance spectra of (a) PM6:D18:L8-BO and (b) PTQ10:L8-BO photoactive layers after 24 h illumination by low light (~0.1 sun) intensity light, compared to fresh samples. The spectra were recorded at ambient conditions for thin organic bulk heterojunction layers deposited on ITO/SnO_2 substrates.

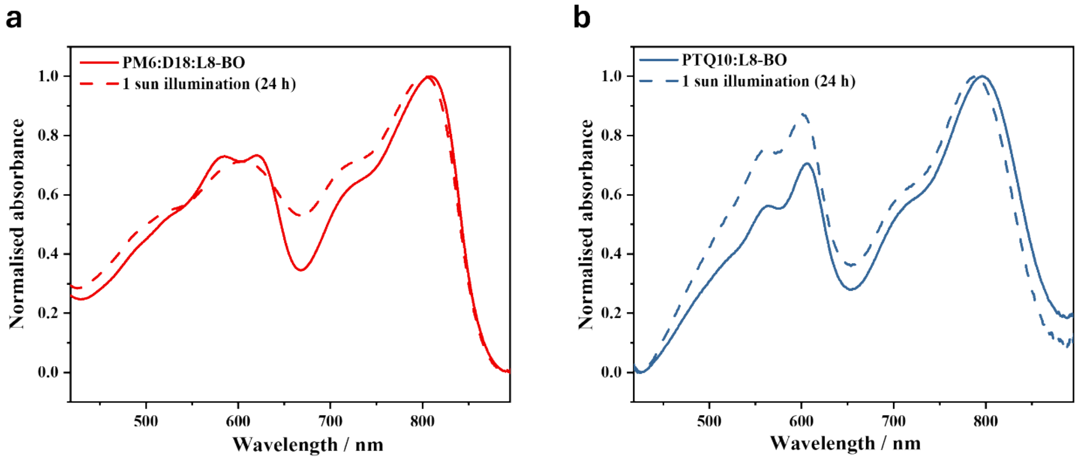

Fig. S8 Normalised UV-Vis absorbance spectra of (a) PM6:D18:L8-BO and (b) PTQ10:L8-BO photoactive layers after 24 h illumination by 1 sun intensity light, compared to fresh samples. The spectra were recorded at ambient conditions for thin organic bulk heterojunction layers deposited on ITO/SnO_2 substrates.

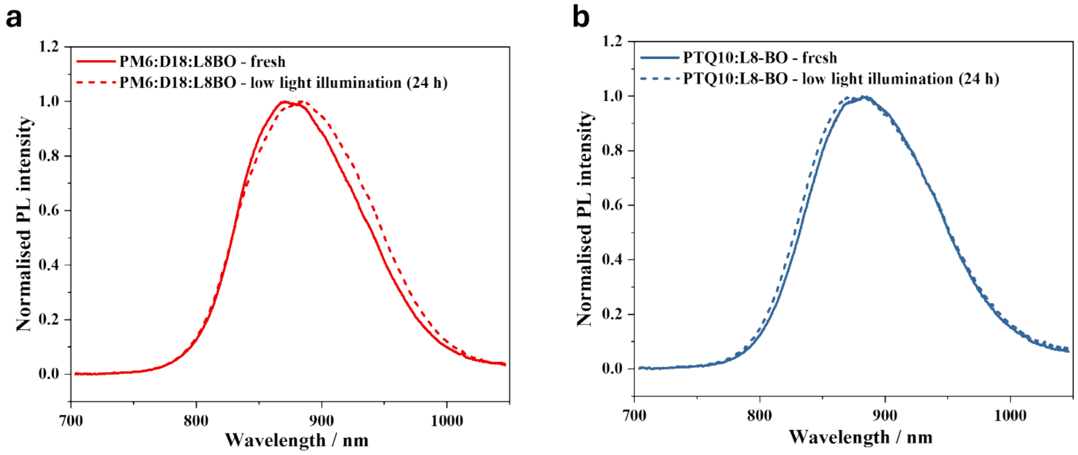

Fig. S9 Normalised photoluminescence (PL) spectra of (a) PM6:D18:L8-BO and (b) PTQ10:L8-BO photoactive layers after 24 h illumination by low light (~0.1 sun) compared to fresh samples. The spectra were recorded at ambient conditions for thin organic bulk heterojunction layers deposited on ITO/SnO_2 substrates.

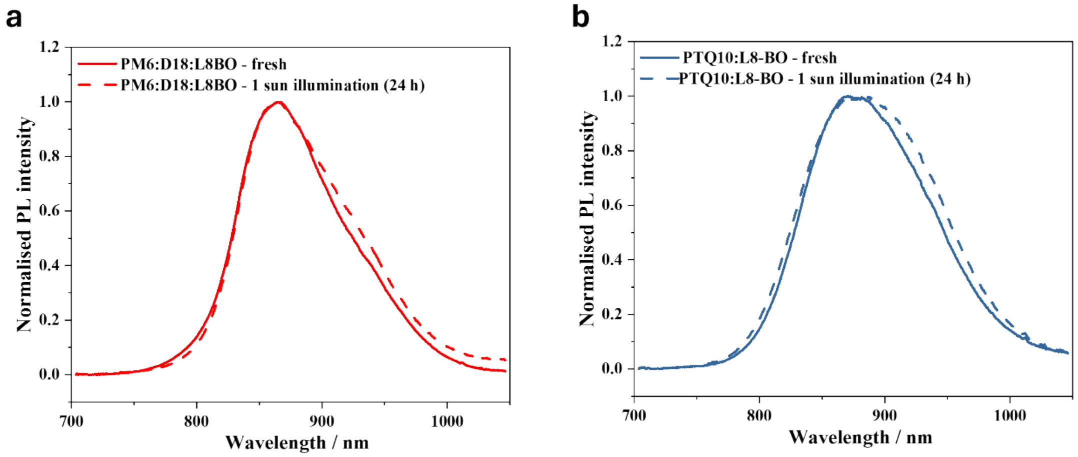

Fig. S10 Normalised photoluminescence (PL) spectra of (a) PM6:D18:L8-BO and (b) PTQ10:L8-BO photoactive layers after 24 h illumination by 1 sun, compared to fresh samples. The spectra were recorded at ambient conditions for thin organic bulk heterojunction layers deposited on ITO/SnO_2 substrates.

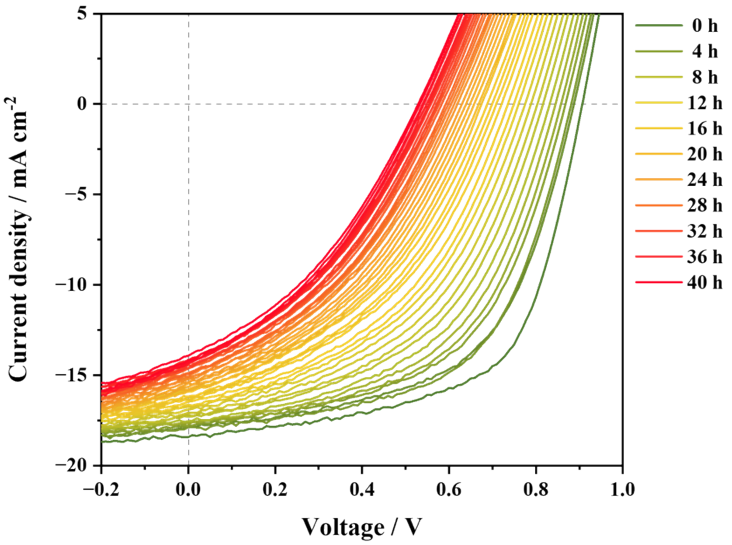

Fig. S11 Operational stability of a PTQ10:L8-BO organic IPV-anode with an active area of 0.2 cm^2 measured as a solar cell for 40 h at ambient conditions under continuous 1 sun illumination. The device was kept at 0.2 V applied bias and current density–voltage scans were recorded once every hour. The horizontal and vertical dashed lines indicate 0 mA cm^{-2} current density and 0 V applied bias, respectively.

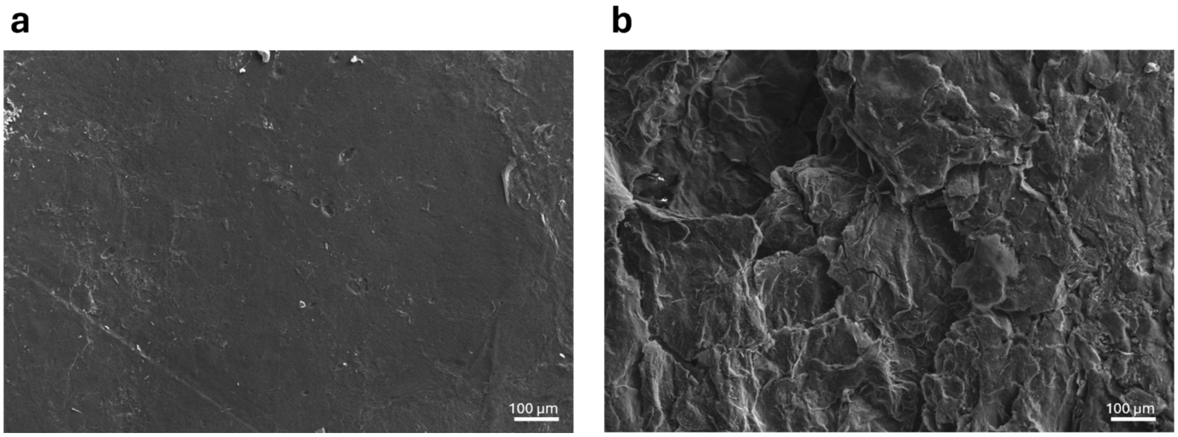

Fig. S12 Top-view scanning electron micrographs of the top 150 µm-thick graphite/NiFeOOH layer of an organic PTQ10:L8-BO IPV-anode before and after 52 h of operational water oxidation stability (at +1.23 V_{RHE} in 1 M NaOH electrolyte under continuous 1 sun illumination).

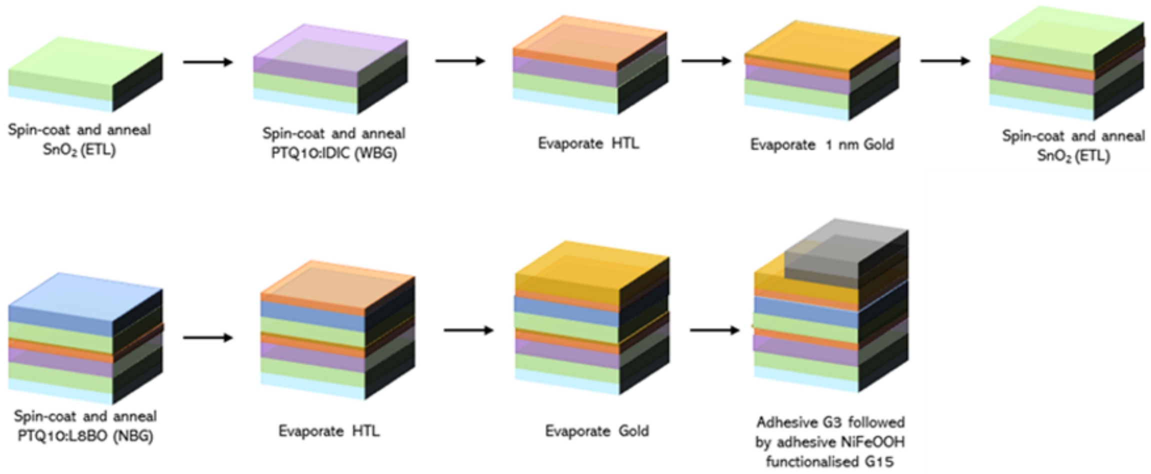

Fig. S13 Schematic illustration of the fabrication steps of monolithic tandem organic IPV-anodes containing wide-bandgap (WBG) PTQ10:IDIC and narrow-bandgap (NBG) PTQ10:L8-BO photoactive layers. Similar steps were followed to fabricate the single-junction PTQ10:L8-BO IPV-anode, except that the first four steps were omitted.

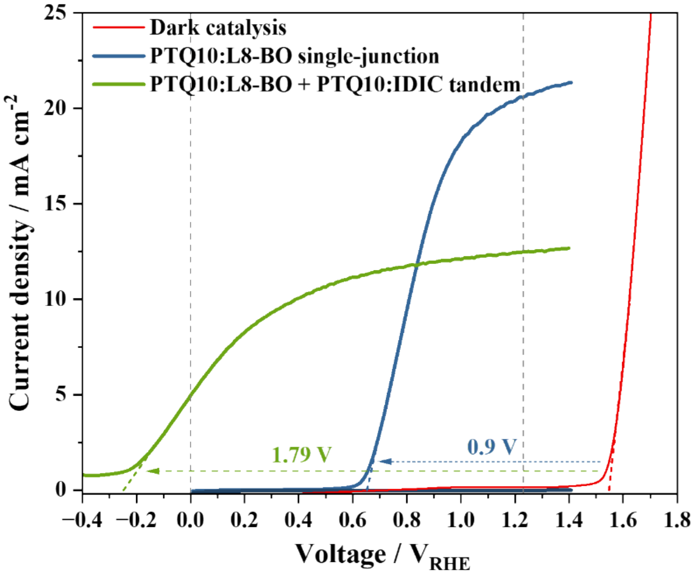

Fig. S14 Performance of single-junction and tandem IPV-anodes incorporating PTQ10:L8-BO and PTQ10:IDIC photoactive layers (with ICL of PEDOT:PSS(BM)/Au(1nm)/SnO_2). Current density–voltage scans at 50 mV s^{-1} scan were recorded under 1 sun continuous illumination for a high-performing IPV-anodes. The onset potential of the IPV-anodes (coloured arrows indicate the shift in onset potential) is compared to the dark current density –voltage scan of a

graphite/NiFeOOH catalytic sheet. The measurements were performed in an aqueous, 1 M NaOH electrolyte using a three-electrode setup.

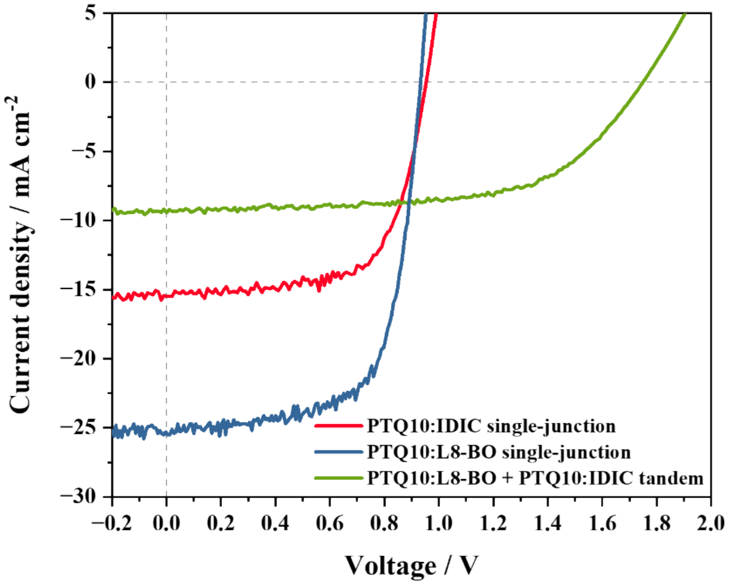

Fig. S15 Current density–voltage scans of solar cells with an active area of 0.05 cm² measured under 1 sun illumination at a scan rate of 1 V s^{-1}. The scans correspond to the various photoactive layers discussed: PTQ10:IDIC as the wide-bandgap layer; PTQ10:L8-BO as both the narrow-bandgap layer in the tandem configuration and the active layer in the single-junction device; and the combination of both layers forming the tandem cell (with ICL consisting of PEDOT:PSS(BM)/Au(1nm)/SnO_2). The high-performing PTQ10:L8-BO single-junction IPV-anode is compared to the monolithic organic PTQ10:L8-BO tandem cell when characterized as photovoltaic devices.

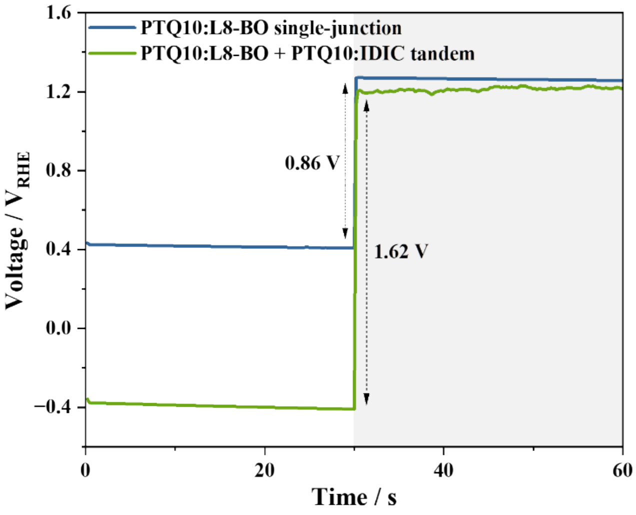

Fig. S16 Open circuit potential of the PTQ10:L8-BO single junction and monolithic tandem (with ICL of PEDOT:PSS(BM)/Au(1nm)/SnO_2) IPV-anodes measured under 1 sun illumination (0 – 30 s, white background) and in dark (30 – 60 s, shaded background) in a three-electrode setup. Dashed vertical lines with arrows indicate the difference in open circuit potential upon switching off the illumination. The measurements were performed in an aqueous, 1 M NaOH electrolyte.

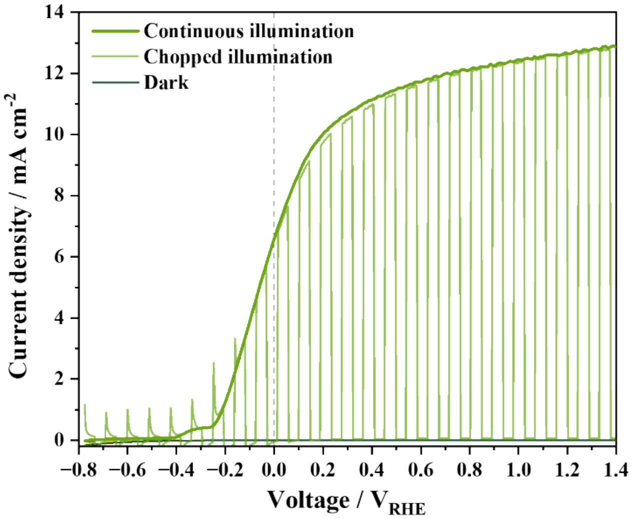

Fig. S17 Current density–voltage scans in dark, and under 1 sun continuous and chopped illumination for a monolithic tandem organic IPV-anode incorporating PTQ10:L8-BO and PTQ10:IDIC photoactive layers (with ICL of PEDOT:PSS(BM)/Au(1nm)/SnO_2). The measurements were performed in a three-electrode setup at 50 mV s^{-1} scan rate in an aqueous, 1 M NaOH electrolyte. Dashed vertical line indicates the applied potential of 0 V_{RHE}.

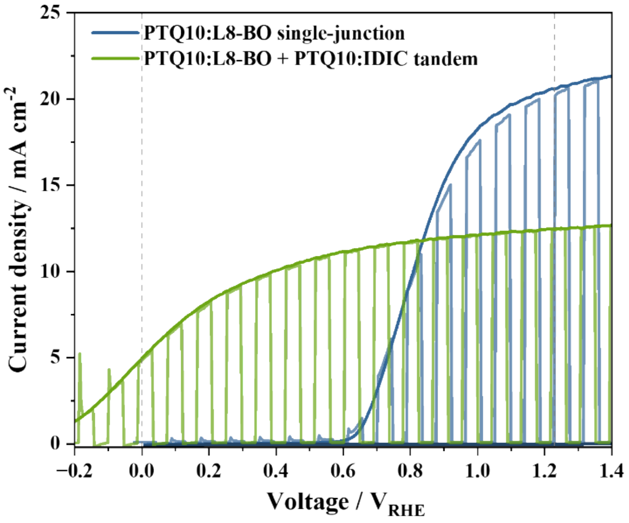

Fig. S18 Performance comparison of single-junction and tandem organic IPV-anodes measured in three-electrode setup. Current density–voltage scans under 1 sun continuous and chopped illumination for a PTQ10:L8-BO single-junction IPV-anode compared to a monolithic organic tandem IPV-anode incorporating PTQ10:L8-BO and PTQ10:IDIC photoactive layers (with ICL of PEDOT:PSS(BM)/Au(1nm)/SnO_2). Dashed vertical lines indicate the applied potential of +1.23 V_{RHE} (to the right) and of 0 V_{RHE} (to the left). The measurements were performed at 50 mV s^{-1} scan rate in an aqueous, 1 M NaOH electrolyte.

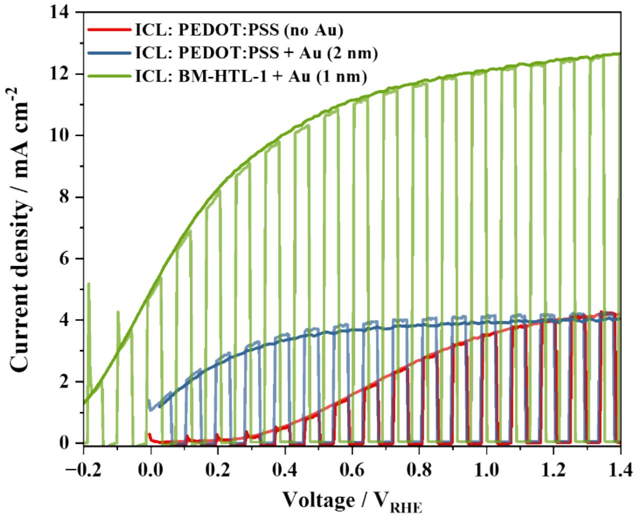

Fig. S19 Current density–voltage scans under 1 sun continuous and chopped illumination of monolithic tandem organic IPV-anodes incorporating PTQ10:L8-BO and PTQ10:IDIC photoactive layers and different ICLs, namely 2PACz-Br/PEDOT:PSS/SnO_2, 2PACz-Br/PEDOT:PSS/Au/SnO_2, and PEDOT:PSS(BM)/Au(1nm)/SnO_2. The measurements were performed in a three-electrode setup at 50 mV s^{-1} scan rate, in an aqueous, 1 M NaOH electrolyte.

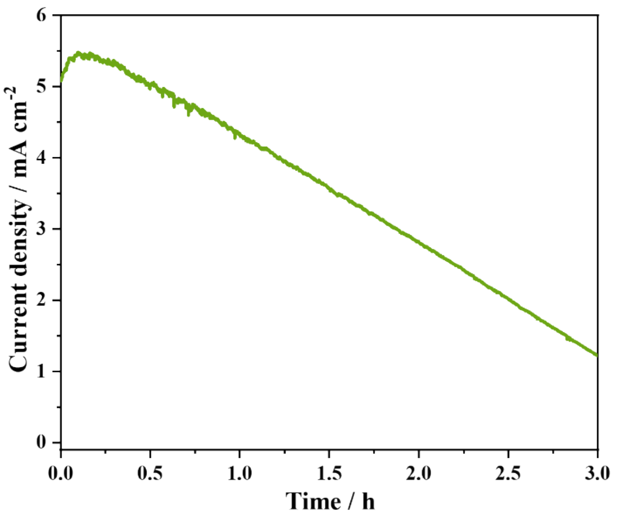

Fig. S20 Bias-free operational solar water splitting stability of the monolithic tandem IPV-anode incorporating PTQ10: L8-BO and PTQ10:IDIC photoactive layers (with ICL of PEDOT:PSS(BM)/Au(1nm)/SnO_2. The measurement was performed in a two-electrode setup in an aqueous, 1 M NaOH electrolyte.

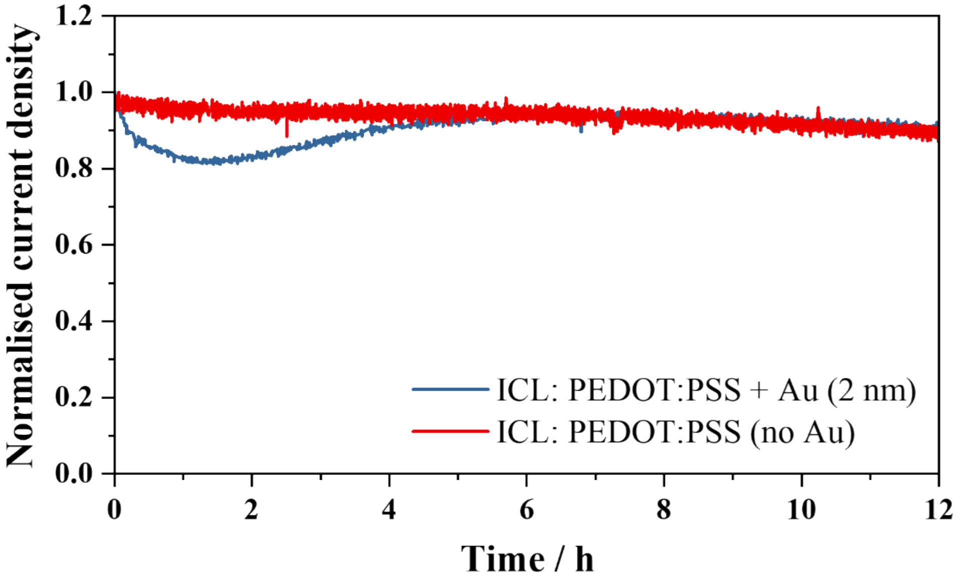

Fig. S21 Operational photocurrent stability of a PTQ10:L8-BO monolithic tandem organic IPV-anodes with different ICLs, namely PEDOT:PSS (Ossila)/SnO_2, and PEDOT:PSS(Ossila)/Au(2nm)/SnO_2, measured in a three-electrode cell for 12 h at +1.23 V_{RHE}. The measurements were performed in an aqueous, 1 M NaOH electrolyte.

Table S1 Performance of the reference PM6:D18:L8-BO and the PTQ10:L8-BO solar cells. The radiative limit ($V_{oc,rad}$) and corresponding non-radiative voltage losses ($\Delta V_{oc,nrad}$) were calculated using EL and EQE spectra and the reciprocity relation.

	J_{sc} (mA cm^{-2})	FF	V_{oc} (V)	PCE (%)	$V_{oc,rad}$ (V)	$\Delta V_{oc,nrad}$ (V)
PM6:D18:L8-BO	25.3	0.66	0.88	14.7	1.15	0.24
PTQ10:L8-BO	25.4	0.69	0.93	16.3	1.12	0.19

Table S2 Preparation method and conditions of different active layers developed in this work for organic IPV-anodes.

Active layer	Weight ratio	Concentration (mg/mL)	Heat Temperature (°C)	Heating/stirring time (h)
PTQ10:L8-BO	1:1.2	20	50	2.5
PTQ10:IDIC	1:1.5	30	50	1
PM6:D18:L8BO	0.8:0.2:1.2	14	50	2
